# Polyclonal gammopathy after BKV infection in HSCT recipient: a novel trigger for plasma cells replication?

**DOI:** 10.1186/s12985-015-0254-z

**Published:** 2015-02-13

**Authors:** Natalia Maximova, Antonio Pizzol, Aurelio Sonzogni, Massimo Gregori, Marilena Granzotto, Paolo Tamaro

**Affiliations:** Bone Marrow Transplant Unit, Institute for Maternal and Child Health IRCCS Burlo Garofolo, Via dell’Istria 65/1, 34134 Trieste, Italy; Pediatric Hemato-Oncology Department, Institute for Maternal and Child Health IRCCS Burlo Garofolo, Trieste, Italy; Department of Pathology, Ospedale Beato Papa Giovanni XXIII, Bergamo, Italy; Department of Radiology, Institute for Maternal and Child Health IRCCS Burlo Garofolo, Trieste, Italy; Laboratory Department, A.O.U. Ospedali Riuniti, Trieste, Italy

**Keywords:** BKPyV, HSCT, Polyclonal gammopathy, Polyomavirus-associated nephropathy

## Abstract

**Background:**

BK polyomavirus infects most of the general population. However, its clinical manifestations are almost exclusively seen in immunocompromised patients, particularly in kidney and hematopoietic stem cell transplantation recipients.

**Case presentation:**

A 15-y-old female suffering from common B-cell acute lymphoblastic leukaemia underwent hematopoietic stem cell transplantation. The patient had reactivation of BKPyV infection and developed an haemorrhagic cystitis. Three months after transplant, BKPyV viremia and viruria increased and she developed a severe nephropathy associated to a polyclonal gammopathy with high levels of isolated IgM.

**Conclusion:**

This case report describes a rare and unexpected polyclonal gammopathy developed during a polyomavirus-associated nephropathy confirmed by immunohistochemical and laboratory analyses.

## Background

BK virus is a polyomavirus (BKPyV) that infects most of the general population, with seroprevalence rates approaching 90% by 10 years of age [1]. After primary infection, the virus remains quiescent in the uroepithelial cells of kidneys and bladder [2]. Clinical manifestations of BKPyV infection are almost exclusively seen in immunocompromised patients, particularly in those who underwent kidney or hematopoietic stem cell transplantation (HSCT) [3,4].

BKPyV reactivation after allogeneic HSCT manifests at different clinical levels, from asymptomatic viruria to severe hemorrhagic cystitis (HC) [5]. HC occurs in 5% to 68% of HSCT recipients [6,7] and can be associated with significant morbidity and mortality as well as acute kidney injury by obstruction of the urinary tract due to the formation of clots.

BKPyV loads can be measured in urine and serum by quantification of viral DNA with polymerase-chain-reaction (PCR) test [8].

In the era of cyclosporine-based immunosuppression, BKPyV-associated nephropathy had no real clinical significance; conversely, the introduction of third generation immunosuppressors, such as mycophenolate mofetil (MMF) and tacrolimus (FK-506) into routine management of transplant patients has led to the current high prevalence of BKV-related nephropathy [9]. It has been hypothesized that the diversity in BKPyV reactivation risk between cyclosporine and FK-506 could in part be due to the different antiviral activity of the immunosuppressants. Indeed, the in vitro antiviral activity of cyclosporine against several viruses has been demonstrated and its potential effect on BKPyV explored [10].

It has been shown that the most effective treatment for BKPyV infection is the reduction of immunosuppressive therapies. Moreover, the addition of the antiviral agent cidofovir resulted to be helpful in the setting of persistent BK viremia/viruria [11].

This case report shows the development of a rare polyclonal gammopathy in a HSCT patient with a severe nephropathy related to an aggressive BK virus disease.

To the best of our knowledge, this is the first report of a polyclonal gammopathy with lympho-plasma cells infiltration in the renal parenchyma generated from a BKPyV infection.

## Case presentation

A 15-y-old female, suffering from common B-cell acute lymphoblastic leukemia (ALL) with t(1;19)translocation was enrolled in a pediatric protocol AIEOP-BFM LLA 2009. After an initial period of remission, she relapsed during the maintenance phase. Leukemia was consequentely classified as high risk and the patient was treated with AIEOP REC2003 protocol (Italian pediatric protocol for ALL relapsed, LLA REC 2003).

Since chemotherapy alone was considered insufficient and unsafe, bone marrow transplant was performed. The myeloablative conditioning regimen consisted of fractionated doses of total body irradiation (total dose 12 Gy), thiotepa (300 mg/m^2^), cyclophosphamide (1800 mg/m^2^) and rabbit antithymocyte globuline (2.5 mg/kg/day), followed by infusion of non-manipulated bone marrow HSC from HLA matched unrelated donor (MUD). Graft-versus-host disease (GVHD) prophylaxis was performed with FK-506 and MMF.

Quantitative RT- PCR for BKPyV detection was carried out by using the Euro-RT BKV kit (Eurospital, Trieste, Italy), which amplifies a region localized in BKPyV antigen-T gene. Pre-transplant screening for BKPyV was positive resulting in a viremia of 8.3 × 10^4^ cp/ml and a viruria 1.9 × 10^10^ cp/ml, without any clinical signs of infection. PCR screening tests for other virus (CMV, EBV, VZV, HSV, HHV-6, Adenovirus, TTV and JCV) were otherwise negative.

At day +15, neutrophil and platelet engraftment occurred regularly, while lymphocytes were still depleted with only 0.4% out of 600 total WBC. BKPyV viral blood load rose up to 4.4 ×10^5^ cp/ml, with a simultaneous mild decrease of viruria (1.8 × 10^9^ cp/ml). At day +20, the patient developed haemorrhagic cystitis with a rapid exacerbation of symptoms, ureteric bilateral occlusion and, consequently, dilatation of calico-pyelic ducts.

At day +24, absolute lymphocyte count was 11 cell/μl and immunophenotyping showed a predominant CD3^−^CD16^+^/56^+^ cell population (92%), a very low percentage of CD3^+^ T cells (2%), distributed between T CD3^+^CD4^+^ cells (1%) and T CD3^+^CD8^+^ cells (1%) and almost complete depletion of CD19^+^ B lymphocytes (0.6%).

Since cidofovir, one of the few drugs with proven efficacy against BKPyV infection, was unavailable, immunosuppressive therapy was reduced trying to control the disease.

At day +90, two months after immunosuppression reduction, the patient’s bone marrow biopsy revealed the presence of 5% B-lymphoblasts, confirming the morphological relapse of leukemia. On the contrary, there was complete remission of hemorrhagic cystitis, both BKPyV viruria and viremia were unchanged and cytometric analysis showed a severe T and B lymphocyte depletion (CD3^+^ 7%, CD19^+^ 0.1%, absence of CD20, NK cells 91%, among 786 total lymphocyte/μl), confirming a delay of bone marrow reconstruction after HSCT.

At day +107, IgM abruptly increased up to 185 mg/dl (Table 1). Patient’s serum *k* free light chains measured 160 mg/L (ref. val. 6.7-22.4 mg/L) and λ free light chains 211 mg/L (ref. val. 8.3-27.0 mg/L). The ratio κ/λ was 0.76 (ref. val. 0.31-1.56). Urinary protein electrophoresis showed mixed proteinuria with predominance of tubular components. U-Albumin was 5.09 mg/dL (ref. val. <3), U-alfa1 microglobulin 13.3 mg/dL (ref. val. < 1.2) and U-IgG 0.944 (ref. val. < 0.85). Testing for urinary Bence Jones protein was positive: *k* light chains were 5.73 mg/dL and λ light chains 2.89 mg/dL.

**Table 1:** **Levels of immunoglobulin and blood and urinary BK virus in HSCT recipient**

**Day post transplant**	IgG* mg/dl	**IgA mg/dl**	**IgM mg/dl**	**BKV viruria (cp/ml)**	**BKV viremia (cp/ml)**
Pre-BMT	320	4	2	1,9×10^10^	8,3×10^4^
+15	305	3	0	1,8×10^9^	4,4×10^5^
+94	1324	9	15	4,5×10^10^	1,9×10^6^
+98	1166	8	23	3,0×10^9^	4,2×10^5^
+107	784	6	185	8,0×10^8^	3,7×10^5^
+114	1009	5	304	8,2×10^7^	1,1×10^5^
+125	882	7	393	7,9×10^7^	5,4×10^3^
+127	617	5	269	1,9×10^8^	2,0×10^4^
+134	845	7	189	9,0×10^7^	2,9×10^4^
+142	960	123	180	2,0×10^7^	2,0×10^5^
+150	657	70	248	Not performed	Not performed

The table shows IgG, IgM, IgA levels after BKV reactivation, three months after transplantation in a 15-y-old female suffering from common B-cell acute lymphoblastic leukemia. IgM levels rise concurrently to BKV viremia and viruria. *IgG immunoglobulins therapy has been administered.

The hypothesis of lymphoproliferation as a consequence of viral infection was taken into account due to the delay of T-cells reconstruction. Therefore, patient’s blood samples were analyzed by quantitative PCR for the presence of EBV, CMV, Adenovirus, HSV-1, HSV-2, HHV-6, HCV-RNA, HBV-DNA and resulted negative. A leukemic gammopathy was then suspected. A further evaluation of blast immunophenotype did not show significant differences compared to the onset of the disease. Moreover, using flow cytometric analysis, leukemic lymphoproliferative disease was ruled out since no monoclonal B cells were present and, at the same time, there was no evidence of plasmacytosis, neither in peripheral blood nor in bone marrow samples. We also excluded polyclonal gammopathy caused by autoimmune diseases. All serological tests resulted negative (antinuclear antibodies, anti–doublestranded DNA and anti-Smith antibodies, antineutrophil cytoplasmic antibody, anti–glomerular basement membrane antibodies, complement levels of C3 and C4, rheumatoid factor).

**Figure 1:**
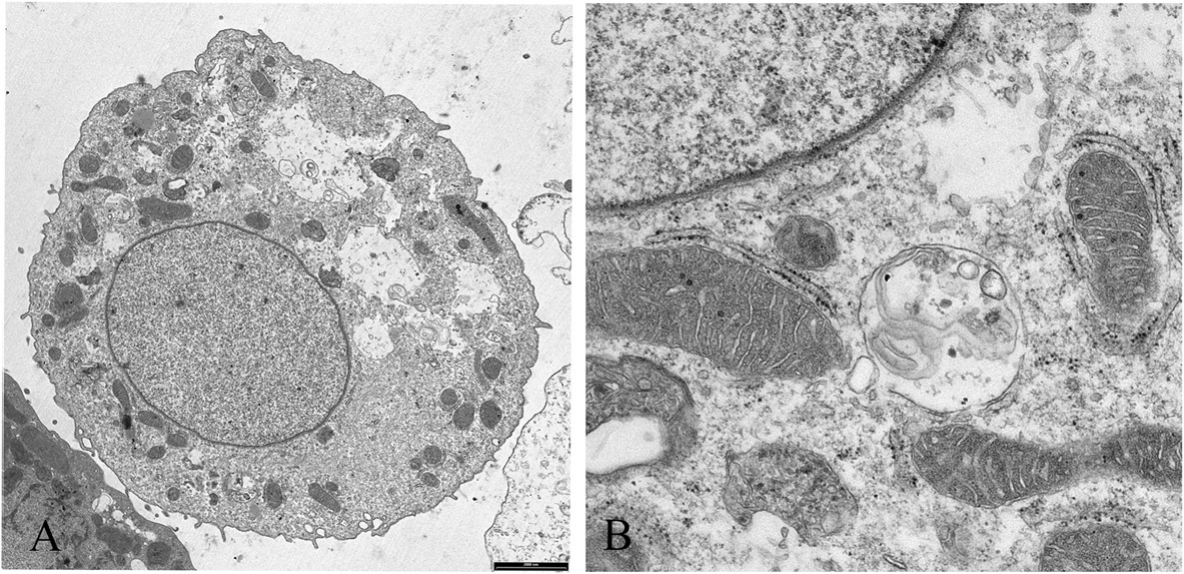
**Electron micrograph of polyomavirus-infected uroepithelial cells of the patient.** Big clusters of viral particles in the nucleus are shown (1000 nm) **(A)**; the organelles have started to degrade, the cytoplasm is homogenous with virus lining the plasma membrane. (Detail, 500 nm) **(B)**.

After few days the patient’s renal function worsened, while urinary cytology showed abundant Decoy cells and big clusters of viral particles in the nucleus of uroepithelial cells were detected by electron microscopy (Figure 1).

**Figure 2:**
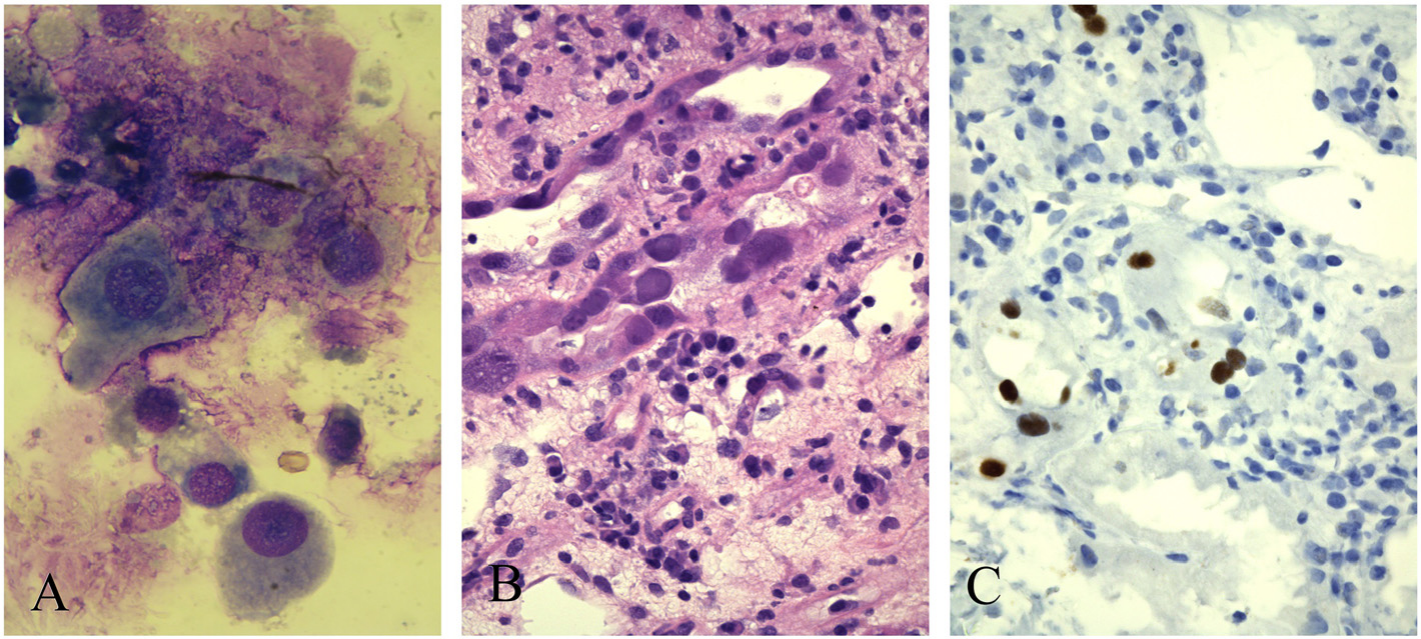
**Abnormal urine cytology with decoy cells.** (May-Grundwald-Giemsa) **(A)**; diffuse inflammatory infiltration of interstitial medullary area represented by lymphocytes, granulocytes and plasma cells with features of epithelial destruction, marked irregularity and hyperchromasia of epithelial cells nuclei (H&E, 100X) **(B)**; positive immunostaining for SV40 large T-antigen antibody in few nuclei of tubular epithelial cells (100X) **(C)**.

A subsequent kidney biopsy showed a diffuse inflammatory infiltration of the interstitial medullary area mainly represented by plasma cells associated with edema without signs of immaturity. Tubular epithelium showed moderate-to-severe atrophy and lesions due to viral cytopathic effect. Confirmation of the identity of the virus was made with immunohistochemical detection of SV40 T-antigen (Figure 2).

**Figure 3:**
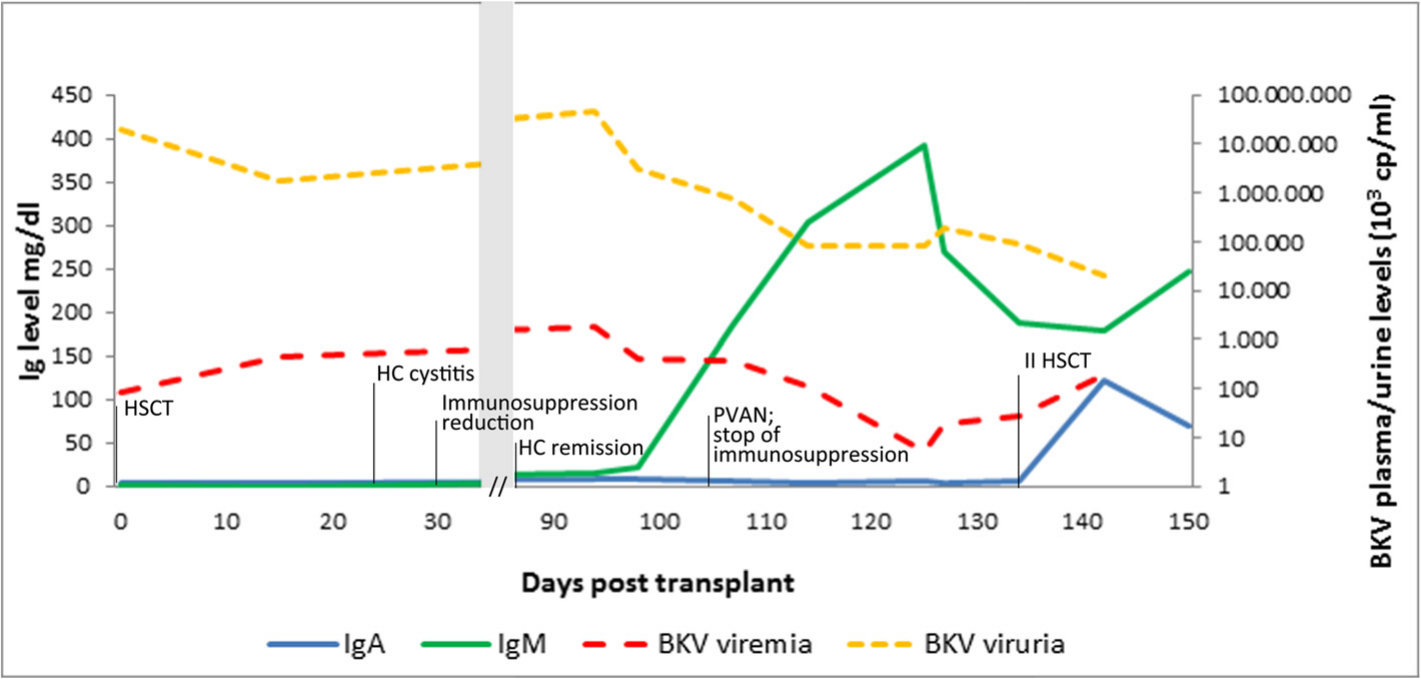
**Values of BKV’s levels in both blood and urine and concomitant Immunoglobulins response.** Figure also shows critical events in chronological subsequence. HC, hemorrhagic cystitis; HSCT, hematopoietic stem cell transplantation; PVAN, polyomavirus BK-associated nephropathy.

The diagnosis of polyomavirus BK-associated nephropathy (PVAN) was then made and the immunosuppression therapy was discontinued. Despite that, renal function did not improve after the complete interruption of immunosuppressive therapies and viremia and IgM levels remained high (Table 1 and Figure 3).

At day +129 the patient underwent another myeloablative conditioning in order to perform a second HSCT from an haploidentical family donor. At day + 135 bone marrow biopsy showed complete aplasia with no blast cells. IgM levels slowly decreased, probably due to the complete depletion of B cells induced by myeloablative conditioning. Unfortunately, as BKPyV's viremia rose back to high levels as well as the immunoglobulins and eventually, three weeks after the second HSCT the patient died. Death was ascribable to a multiple organ failure due to chemotherapy toxicity after second transplant conditioning regimen, rather than just PVAN.

## Conclusion

According to Kidney Disease Improving Global Outcome Clinical Practice Guidelines for the Care of Kidney Transplant Recipients and the guidelines of the American Society for Transplantation Infectious Disease Community of Practice, polymerase chain reaction for BKPyV DNA in plasma is recommended for screening and diagnosis of PVAN. PCR of urine, urinary cytology, and electron microscopy of urine are also described as optional examinations. The definitive diagnosis of PVAN should be sought by demonstrating PV cytopathic changes in renal tissue, and confirmed by immunohistochemistry or in situ hybridization (proven PVAN) [12].

During the last three years, BKPyV reactivation has been weekly monitored in transplant patients of our Transplant Unit and both viremia and viruria incidence were in accordance to previous reported values of 20% and 60%, respectively. On the contrary, it has been noticed that absolute levels of viral blood load were higher than expected (> 10^4^ copies/ml) leading to the development of more significant renal function impairment compared to those with low viremia, as it has been reported in the setting of patients who underwent renal transplant [13].

The onset of PVAN in those who underwent HSCT is rare when compared to BKPyV related haemorrhagic cystitis, that occurs in up to 25% of patients [4]. There are only few PVAN reported cases with proven biopsy following allogeneic HSCT [11,14-16].

The treatment of PVAN is a challenge for physicians. Since clinical manifestations of BKPyV infection were evident in our patient and cidofovir was unavailable at that time, the only possible therapeutic choice was immunosuppressive therapy reduction. Moreover, bone marrow biopsy, performed at day +32, was positive for minimal residual disease (MRD): in this case, the suspension of the immunosuppressive therapy could allow an improvement in the T cell-mediated immunity against the virus with an effect of Graft versus Leukemia (GvL) in order to limit or retard leukemia relapse. Unfortuntely, there was no T cell-mediated immunity amelioration. The patient remained T-depleted until second transplantation was performed.

It is well known that HSCT recipients have a profound impairment of the immune function during the first four or five months post-grafting, regardless of the type of graft (autologous, syngeneic, or allogeneic), the type of underlying disease, the conditioning regimen, the type of post-grafting immunosuppression, the preparative regimen or the presence of acute GVHD [17]. This leads to a condition of hypo-immunoglobulinemia that could require also immunoglobulin replacement therapy. Thus, the development and maturation of B cells can be significantly altered in the post-transplant period, especially in the setting of ongoing immunosuppression or chronic GVHD. Normal levels are generally reached within 6–12 months.

It has been observed that isolated episodes of increased levels of IgM or, rarely, IgA in the early post-transplant period could happen, even if this is not related to viral infections or other relevant complications. These observations are confirmed by literature reports on the development of clonal gammopathies after myeloablative allogenic or autologous transplantation [18-20] non related to malignancy, EBV reactivation or autoimmune diseases. A possible explanation could be the immune post-HSCT dysregulation, characterized by a delay in the reconstitution of B- and T-cell lineages [21]. In this case report, the patient developed hyper-IgM during polyclonal gammopathy. Clonal gammopathy onset related to herpes viruses (EBV and/or CMV) is well known and potentially life-threatening in patients who have performed HSCT, especially if there is a progression to PTLD. Anyway, there are no reports about the relation between polyclonal gammopathy and PVAN. As far as we know, BKPyV has no lymphoproliferative activity, unlike EBV or others herpes viruses.

The type of inflammation in PVAN was almost purely mononuclear, consisting mainly of plasma cells. Focal or diffuse interstitial aggregates of plasma cells between tubules has been reported in 50% of index biopsies in kidney transplants [22]. Moreover, Kemeny et al. showed that in plasma cell-rich (PR) biopsies, plasma cells predominantly produce IgM, and that patients with PVAN have significantly higher serum IgG and IgM activities than patients without PVAN.

Rhandawa et al. have studied the humoral immune response to BK virus infection in renal transplants showing that among patients with paired serum samples, most of those with BKPyV viruria or viremia displayed a substantial increase in IgG, IgA and IgM antibodies levels, indicating that transplant recipients are capable of mounting anamnestic antibody responses to BKPyV [23].

There are no reports concerning the relationship between PR biopsy in PVAN with mono- or polyclonal gammopathies, as reported in our case. It is well known that in HSCT recipients after myeloablative conditioning and whereas full-donor chimerism has been proven, immunoglobulin production totally depends from donor cells.

Considering that in kidney recipients BKPyV serum antibodies may partly derive from local intragraft production and that serum antibody levels can also reflect the induced immunological response in lymphoid organs [13], we can hypothesize that, in the reported patient, BKPyV infection could have led to an inflammatory-induced dysregulation of the donor primitive plasma cells.

Rarely, in the post-transplant period, there are pathological high levels of immunoglobulins, especially in patients with complete lymphocyte depletion. Lymphoproliferative disease due to viral infections as EBV or CMV must first be suspected. Anyway, our case suggests that in the presence of renal impairment, BKyV infection should be evaluated and viral tests for BKPyV viremia and viruria should be performed especially in those patients in which BKPyV has not been routinely screened. Indeed, BKPyV has acquired more and more important roles in HSCT recipients and this is even more evident after the introduction of FK-506 and MMF as prophylaxis for GVHD.

### Ethics and consent

Research Ethics approval for the publication of this case report was obtained from the Ethics Committee of the our Institute. Written informed consent for the publication of case report and accompanying images and data was obtained from the parents of the patient. A copy of the written consent is available for review by the Editor of this journal. All methodology reported in this paper served for the sole purpose of diagnostics. The study has been approved by the Institutional Review Board of the Institute for Maternal and Child Health IRCCS “Burlo Garofolo”.
